# Comparison of viromes in vaginal secretion from pregnant women with and without vaginitis

**DOI:** 10.1186/s12985-020-01482-z

**Published:** 2021-01-06

**Authors:** He-Teng Zhang, Hao Wang, Hai-Sheng Wu, Jian Zeng, Yan Yang

**Affiliations:** 1 Department of Obstetrics and Gynecology, The Fourth Affiliated Hospital of Jiangsu University, 20 Zhengdong Road, Zhenjiang, 212001 Jiangsu China; 2 School of Medicine, Jiangsu University, Zhenjiang, 212013 Jiangsu China; 3 Department of Clinical Laboratory, Huai’an Hospital, Xuzhou Medical University, Huai’an, 223002 Jiangsu China; 4 Qinghai Institute for Endemic Disease Prevention and Control, Xining, 810021 Qinghai China

**Keywords:** Pregnant women, Vaginitis, Virome, Human papillomavirus, Anellovirus, Norovirus

## Abstract

**Background:**

Although some studies have investigated the bacterial community in vaginal tract of pregnant women, there are few reports about the viral community (virome) in this type of microenvironment.

**Methods:**

To investigate the composition of virome in vaginal secretion samples, 40 vaginal secretion samples from pregnant women with vaginitis and 20 vaginal secretion samples from pregnant women without vaginitis, pooled into 4 and 2 sample pools, respectively, were subjected to viral metagenomic analysis.

**Results:**

Results indicated virus sequences showing similarity to human papillomavirus (HPV), anellovirus, and norovirus were recovered from this cohort of pregnant women. Further analysis indicated that 15 different defined types and one unclassified type of HPV were detected from pregnant women with vaginitis while only 3 defined types of HPV were detected in pregnant women without vaginitis. Five different groups of viruses from the family *Anelloviridae* were present in pregnant women with but none of them were detected in pregnant women without vaginitis. Norovirus was detected in 3 out of the 4 sample pools from pregnant women with vaginitis but none in the pregnant women without vaginitis. Twelve complete genomes belonging to 10 different types of HPV, and 5 novel anllovirus genomes belonging 2 different genera in *Anelloviridae* were acquired from these libraries, based on which phylogenetical analysis and pairwise sequence comparison were performed. Phageome in these samples was also briefly characterized and compared between two groups.

**Conclusion:**

Our data suggested that virome might play an important role in the progression of vaginitis in pregnant women.

## Introduction

Vaginal microecology is a dynamic balance state of mutual restriction and coordination composed of vaginal microorganisms, normal vaginal anatomy, periodic endocrine changes and local vaginal immune system. It is very important to maintain vaginal health microenvironment by the interaction and balance between microorganisms and hosts. Cervicovaginal environment is considered to be responsible for the development of cervical disease and the infection of high-risk human papillomaviruses (HPVs) [1]. The vaginal microbiota coexists with variety of microorganisms in healthy cervicovaginal environment. Dominant *Lactobacillus* species can decrease the pH value of the cervicovaginal environment, which creates a chemical defense against the infection of exogenous bacteria and viruses [2–4]. Reports have revealed the potential role of elevated pH value on the vaginal microbiota in cervical carcinogenesis [2, 5, 6]. In addition, some studies have indicated that increasing diversity of vaginal microbiota were associated with the progression of cervical intraepithelial neoplasia (CIN) disease and possibly involved in regulating persistent HPV infection [7].

Viral infections are common in vagina and cervix of healthy, asymptomatic women. Some viruses (e.g. HPV and Herpesvirus) were frequently detected in the same subject over time, suggesting these viruses can cause persistent or latent infection in genital tract of women, where the persistent infection with HPV is associated with cervical as well as anogenital cancers [8]. Although many viral pathogens are known to be important components of the vaginal microbiome, viruses have been less studied than bacteria. Two recent reports have investigated the vaginal virome to analyze the link between phageome and bacterial vaginitis [9] and the link between eukaryotic DNA virome and preterm birth [10]. However, the two studies both only investigated the DNA virus in vagina. Here, using viral metagenomics, were investigated and compared the viromes (including eukaryotic virome and phageome) between pregnant women without vaginitis and these with vaginitis.

## Materials and methods

### Sample collection and preparation

For viral metagenomic analysis, 20 and 40 vaginal swab samples were collected from pregnant women without vaginitis and those with vaginitis, respectively, who were admitted to the Department of Obstetrics and Gynecology at the Fourth Affiliated Hospital of Jiangsu University from Jan. 2018 to Jan. 2019 for antepartum examination, with ages of 24 to 37 years old. After sample collection, tips of the swabs were immersed into 0.5 mL PBS and vigorously vortexed for 5 min and incubated for 30 min at 4 °C. 200 µL supernatant of each sample was then collected after centrifugation (10 min, 15,000 × *g*), which were randomly mixed into 6 sample pools, including 4 pools from pregnant women with vaginitis and 2 pools from pregnant women without vaginitis, where each pool included 10 samples. The pooled supernatants were further filtered through 0.45-mm filters (Millipore) to remove eukaryotic- and bacterial cell-sized particles, and 200 µL of supernatant from each pool was then subjected to a mixture of nuclease enzymes to reduce the concentration of free (non-viral encapsidated) nucleic acids [11, 12]. Remaining total nucleic acid was then extracted using QIAamp MinElute Virus Spin Kit (Qiagen) according to manufacturer's protocol.

### Viral metagenomic sequencing and bioinformatic analysis

Six libraries were then constructed using Nextera XT DNA Sample Preparation Kit (Illumina) and sequenced on the MiSeq Illumina platform with 250 bp ends with dual barcoding for each library. For bioinformatics analysis, the raw data generated by MiSeq were de-barcoded using vendor software from Illumina. An in-house analysis pipeline running on a 32-nodes Linux cluster was used to process the data [12]. Reads were considered duplicates if bases 5 to 55 were identical and only one random copy of duplicates was kept. Clonal reads were removed and low sequencing quality tails were trimmed using Phred quality score ten as the threshold. Adaptors were trimmed using the default parameters of VecScreen which is NCBI BLASTn with specialized parameters designed for adapter removal. All of the clean reads > 50 bp were de novo assembled within each barcode using the ENSEMBLE assembler [13]. Contigs and singlets were then subjected to BLASTx searching against a customized viral proteome database with an E-value cutoff of < 10^–5^, where the viral protein database was prepared using NCBI virus reference proteome (ftp://ftp.ncbi.nih.gov/refseq/release/viral/) to which was added viral proteins sequences from NCBI nr fasta file (based on annotation taxonomy in Virus Kingdom). To reduce false positives, candidate viral hits were matched to an in-house non-virus non-redundant (NVNR) protein database using BLASTx searching, where the NVNR database was compiled using non-viral protein sequences extracted from NCBI nr fasta file (based on annotation taxonomy excluding Virus Kingdom). Hits showing a higher (less significant) E score to NVNR than to viral sequences were removed.

### Viral reads distribution and genome acquisition

For viral reads distribution, all the single reads > 50 bp of the clean data of were compared to viral proteome database using BLASTx as mentioned above and the BLASTx results were then loaded into the MEGAN program for viewing profiles and virus type attributes [14]. For genome acquisition of Human papillomavirus (HPV) and anellovirus, BLASTing resulted sequence reads of these 2 types of virus within each library were respectively de novo assembled into contigs using CLC genomics workbench 10.0 with stringent parameters. Only the assembled sequences showed at least 50 bp overlapping at the start and end, confirming their circular genomes, were considered to be putative complete HPV or anellovirus genomes and subjected to further ORF finding.

### Phylogenetic analysis

Phylogenetic analysis was performed based on the complete genome sequences of the HPVs and deduced amino acid sequence of ORFs of anellovirus acquired here, their closest relatives based on BLASTn (for HPV) or BLASTp (for anellovirus), and other representative virus strains in the genus *Alphapapillomavirus* or family *Anelloviridae*. Sequence alignment was performed using CLUSTAL W with the default settings [15]. A phylogenetic tree with 1,000 bootstrap resamples of the alignment data sets was generated using the maximum-likelihood (ML) method in MEGA7.0. Bootstrap values for each node were given [16].

### Study approval

Patients signed informed consent forms, and protocols were approved by Medical Ethical Committee at the Fourth Affiliated Hospital of Jiangsu University (Reference code: ujs5thhos20180301).

## Results

### Viral sequences recovered the 6 vaginal swab sample pools

**Fig. 1:**
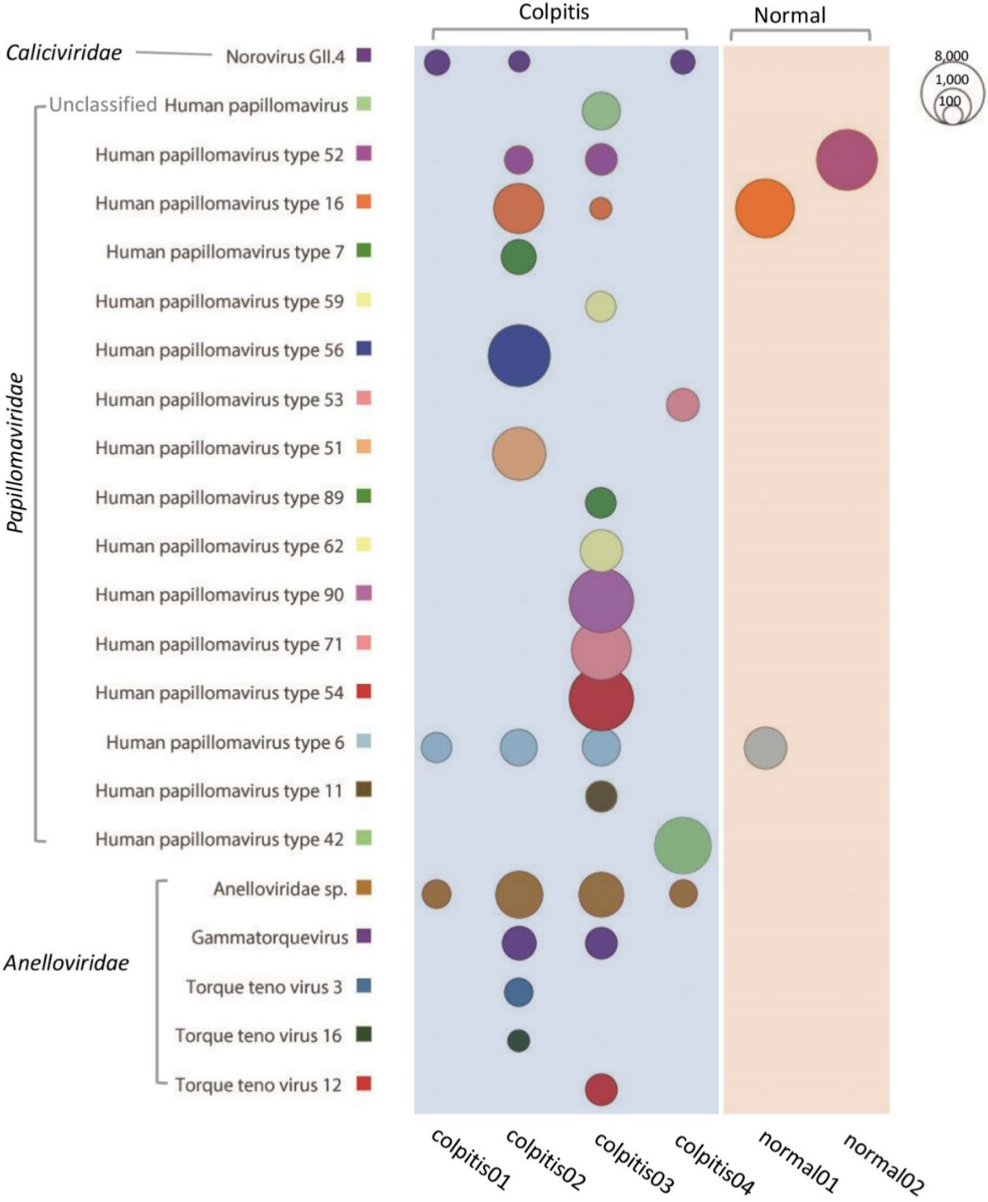
Sequence read distribution of different virus in vaginal tract of pregnant women revealed by viral metagenomics. The counts of sequence reads aligning to different virus are calculated and represented using bubbles. Each bubble represents the log 10 transformed number of viral reads. Species and type of viruses detected in these libraries are labeled at left side and library IDs are labeled under corresponding column

**Fig. 2:**
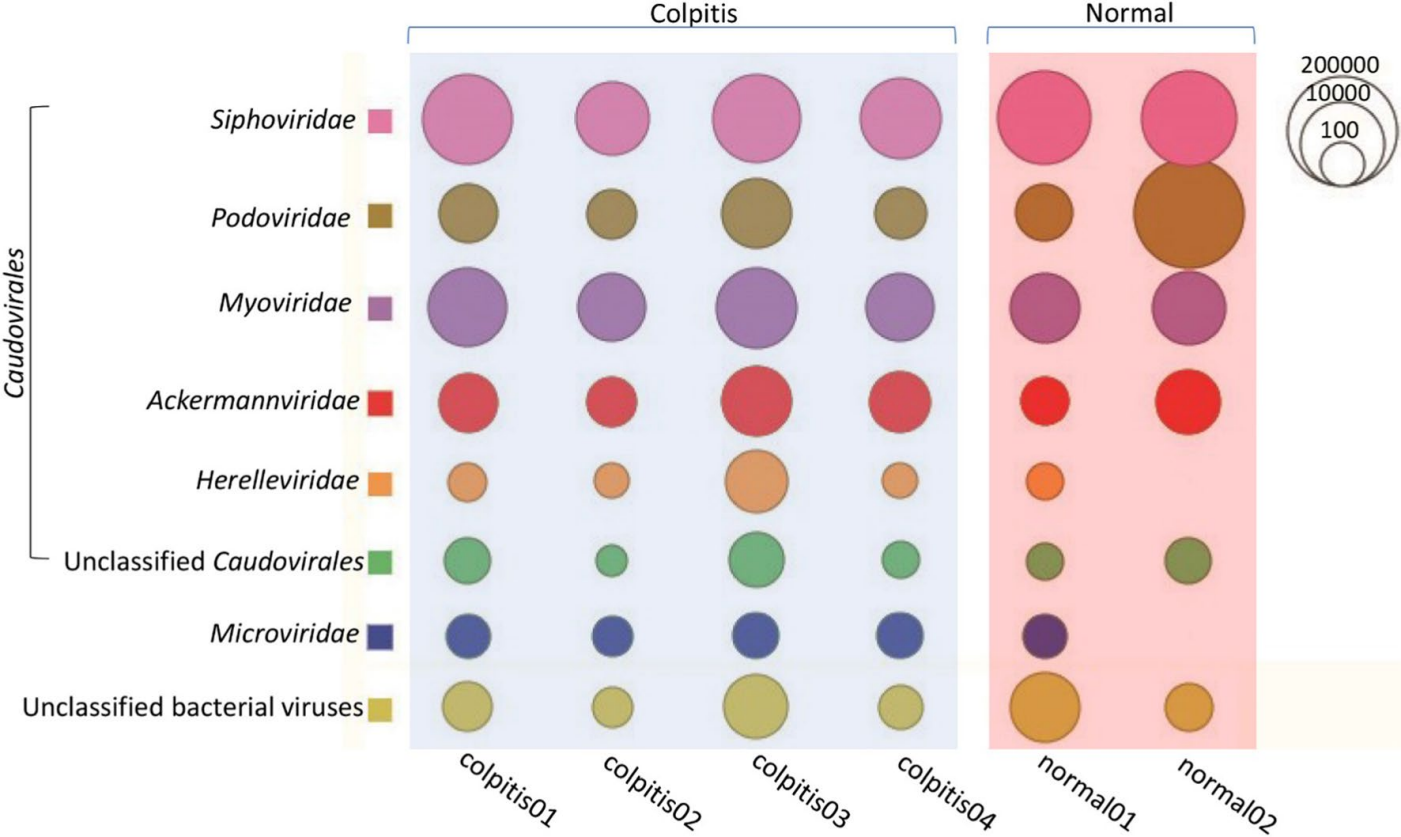
Sequence read distribution of different group of bacteriophages in vaginal tract of pregnant women revealed by viral metagenomics. The counts of sequence reads aligning to different families of phages are calculated and represented using bubbles. Each bubble represents the log 10 transformed number of viral reads. Family names of phages detected in these libraries are labeled at left side and library IDs are labeled under corresponding column

The 60 vaginal swab samples of the 6 libraries generated a total of 13,309,272 sequence reads, which included 8,454,074 from 4 vaginitis libraries and 4,855,198 from 2 non-vaginitis libraries, where 88.6% of the total bases having quality scores > Q30. BLASTx results were further analyzed in MEGAN program, which yielded the different virus types and bacteriophage read distribution in the 6 libraries. Short reads aligning to different species of viruses and bacteriophage were counted and their log 10 transformed values were represented using bubbles (Figs. 1, 2). Results indicated that HPV was the predominant virus detected in all the 6 libraries. A total of 15 different defined and one unclassified HPV types were detected in the samples from the vaginitis group, while only 3 type of HPV were recovered from the samples from non-vaginitis group. Apart from HPV, a large number of anellovirus sequences were also detected in the 4 libraries from pregnant women with vaginitis but in none of the libraries from pregnant women without vaginitis. The anelloviruses detected in the vaginitis swab samples included 3 defined species and two groups of anellovirus belonging to Anelloviridae sp. and unclassified species in the genus *Gammatorquevirus*, respectively. Unexpectedly, 3 of the libraries from vaginitis group also yielded numerous noroviral sequence reads, which were all from type Norovirus GII.4 (Fig. 1). Figure 2 showed the bacteriophage reads distribution in the 6 libraries, which indicated that 6 different families and 2 groups of unclassified phages were detected in these samples. Comparing to these 4 libraries from pregnant women with vaginitis one of the libraries from women without vaginitis(normal2) showed the absence of phages belonging to *Microviridae* and *Herelleviridae*.

### Genomes of HPV and Anellovirus and phylogenetic analysis

**Fig. 3:**
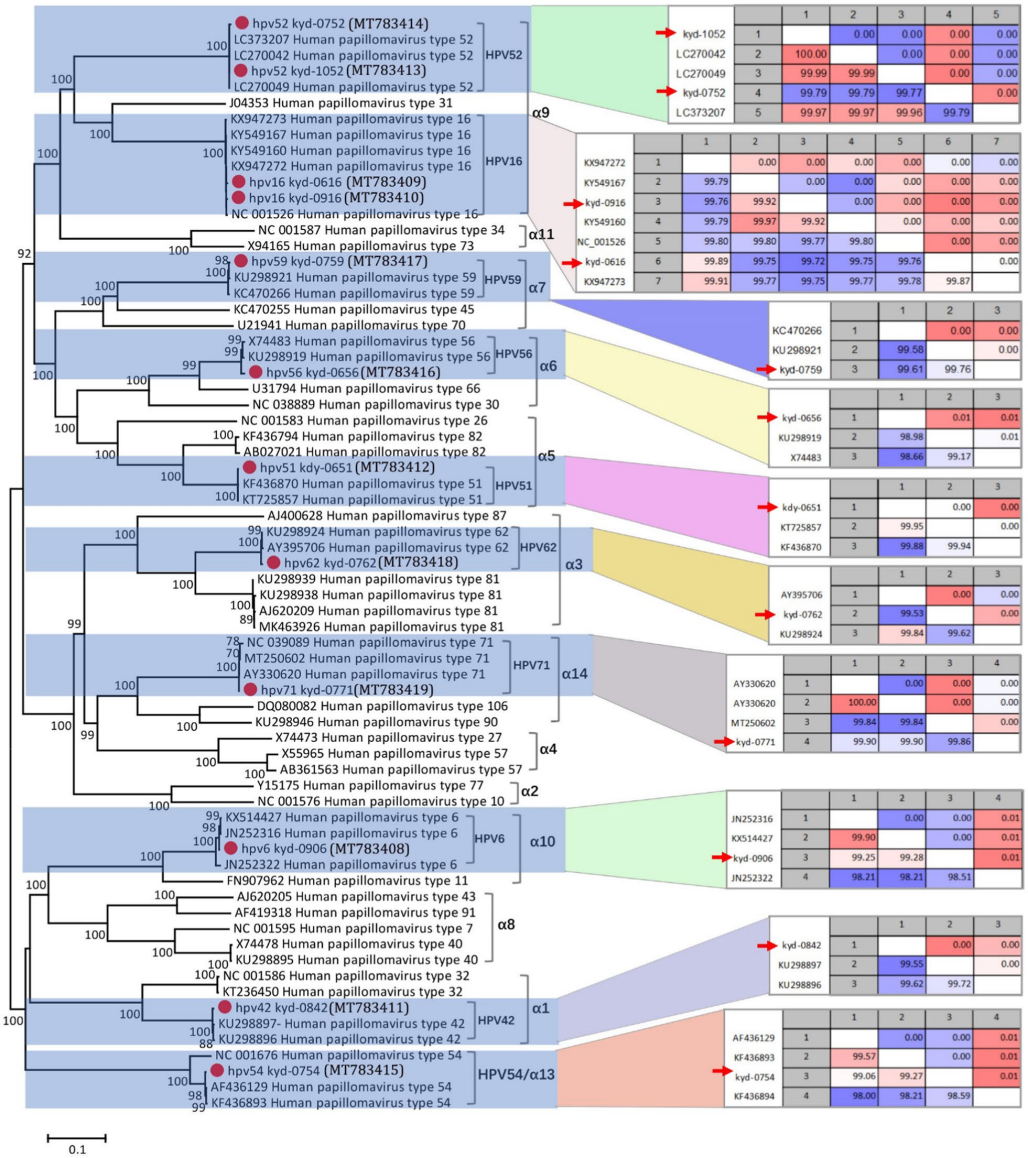
Phylogenetic analysis and pairwise sequence comparison based on the complete genome of HPV. The complete genomes of HPV in the phylogenetic analysis included the 12 complete genomes in this study, their closest relatives based on the BLASTn in GenBank, and other representative types of HPV in genus *Alphapapillomavirus*. The pairwise comparison based on complete genome among different strains within the same type of HPV is shown beside the corresponding clusters. The complete genomes acquired in this study are labeled with red dots

**Fig. 4:**
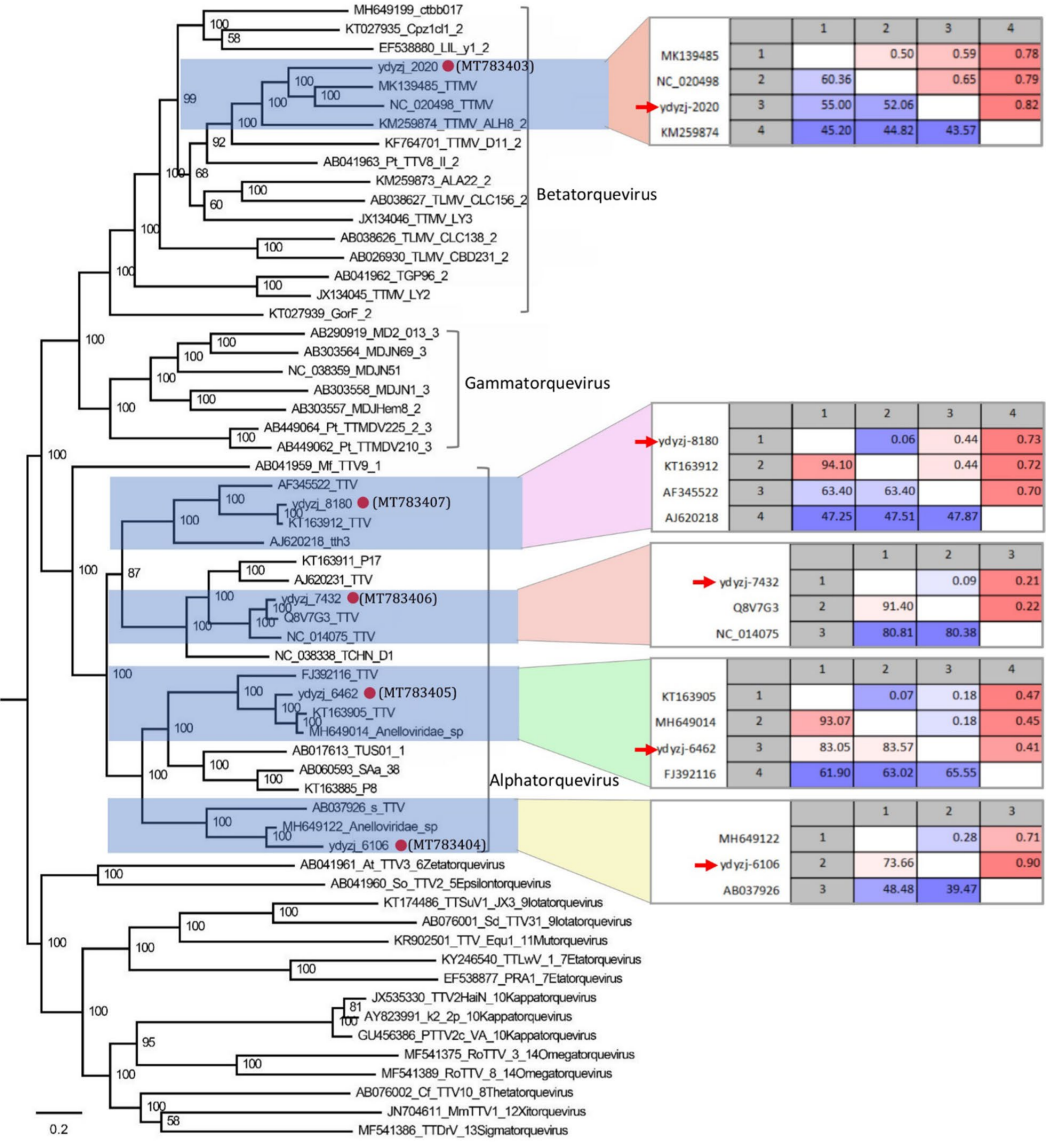
Phylogenetic analysis and pairwise sequence comparison based on the amino acid sequence of anelloviruses discovered in vaginal tract of pregnant women. The amino acid sequence of ORF1 in the phylogenetic analysis included the 5 anllovirus strains in this study, their closest relatives based on the BLASTp of ORF1 protein in GenBank, and other representative species of anellovirus in the family *Anelloviridae*. The pairwise comparison based on ORF1 protein among different strains within the same group of anellovirus is shown beside the corresponding clusters. The anelloviruses identified in this study are labeled with red dots

Twelve complete HPV genomes were generated by de novo assembly of HPV sequence reads in individual libraries, including types of HPV6 (n = 1), HPV16 (n = 2), HPV42 (n = 1), HPV51 (n = 1), HPV52 (n = 2), HPV54 (n = 1), HPV56 (n = 1), HPV59 (n = 1), HPV62 (n = 1), and HPV71 (n = 1). The 12 HPV strains were then subjected to phylogenetic analysis based on the complete genome sequence. The pairwise nucleotide sequence identities and distance between different strains in the same type were shown beside the corresponding cluster (Fig. 3). Based on complete genome sequence, the 12 HPV strains shared 98.51%-100% sequence identities to their closest relatives, suggesting the conserved property of these strains. Five anellovirus genomes were acquired from vaginal swab samples of the pregnant women with vaginitis, 4 of which belonged to the genu *Alphatorquevirus* while the remaining one were from the genus *Betatorquevirus*. Because anellovirus in different genus in the family *Anelloviridae* were too divergent over the nucleotide level, we then established the phylogenetic tree based on the amino acid sequence of ORF1 which is the largest ORF of anellovirus. The pairwise amino acid sequence identities and distances between different strains in the same cluster were shown beside the corresponding site (Fig. 4). Results indicated that although 3 (MT783405- MT783407) of the anelloviruses identified here shared > 80% sequence identities with their best matches (based on BLASTp searching in GenBank), 2 of them (MT783403 and MT783404) shared lower sequence identity with the known anelloviruses available in GenBank, suggesting these two anelloviruses may qualified to be new species or new virus type within *Betatorquevirus* and *Alphatorquevirus*, respectively, especially the strain ydyzj_2020 (MT783403) only had the highest sequence identity of 55.0% with a TTV-like mini virus (TTMV) (GenBank no. MK139485), which is a anellovirus from respiratory tract sample in Vietnam based on the annotation of the sequence in GenBank.

### Nucleotide sequence accession numbers

The 6 sets of raw sequence reads from the 6 viral metagenomic libraries were deposited in the Sequence Read Archive of GenBank database with BioProject accession number: PRJNA645620. The complete genome sequences of human papillomavirus and anellovirus fully characterized in this study were submitted to GenBank with the accession no.: MT783403- MT783419. The GenBank nos. of virus genomes and their corresponding virus strain names and originating library IDs are shown in formation of GenBank no./strain name/originating library ID as follows: MT783408/hpv6_kyd-n0106/normal01, MT783409/hpv16_kyd-n0116/normal01, MT783410/hpv16_kyd-s0216/colpitis02, MT783411/hpv42_kyd-s0442/colpitis04, MT783412/hpv51_kdy-s0251/colpitis02, MT783413/hpv52_kyd-n0252/normal02, MT783414/hpv52_kyd-s0352/colpitis03, MT783415/hpv54_kyd-s0354/colpitis03, MT783416/hpv56_kyd-s0256/colpitis02, MT783417/hpv59_kyd-s0359/colpitis03, MT783418/hpv62_kyd-s0362/colpitis03, MT783419/hpv71_kyd-s0371/colpitis03, MT783403/ydyzj-2020/normal02, MT783404/ydyzj-6106/colpitis03, MT783405/ydyzj-6462/colpitis03, MT783406/ydyzj-7432/colpitis03, and MT783407/ydyzj-8180/colpitis03.

## Discussion

Generally, some researchers focused on the bacterial community in the vaginal microenvironment and their association with bacterial vaginosis which is a common condition characterized with high load of anaerobic micro-organisms and their byproducts that can damage vaginal epithelium and degrade the protective cervical mucus. In bacterial vaginosis anaerobic bacteria disturb the normal balance of vaginal flora causing a 1000 times higher load of bacteria than found normally [17]. This imbalance is associated with scarce peroxidase-producing bacteria (*Lactobacillus*) that normally exist in vaginal milieu, substituted with increased levels of *Gardnerella vaginalis*, *Mycoplasma hominis*, anaerobic *Prevotella bacteroides*, *Porphyromonas* and *Mobiluncus* species [18]. So far there is no reports investigating the viral community in the vaginal microenvironment. Here, we compared the viral community present in the vaginal microenvironment of pregnant women with vaginitis and those in normal conditions. Based on our data, the virus diversity and number of sequence reads in the vaginal swabs from pregnant women are both much higher than those from pregnant women without vaginitis, suggesting viruses may play an important role in the occurrence and development of vaginitis in pregnant women.

The viral community revealed by viral metagenomics in the present study included 3 different groups of viruses including HPV, anellovirus and norovirus. HPV infections are very common and viral DNA can be detected from skin, oral and genital tract from all human populations [19]. So far, over 200 types of HPV have been fully characterized and novel types of HPV are still being found [20–26]. HPV infection may be clinical, subclinical, or latent, which can be high- or low-risk, with high-risk infections having tumorigenic potential. Some HPV infections can also contribute to vaginitis and cervicitis, for example, a report showed that significant increase in HPV prevalence had association with increased severity of cervicitis, cervical intraepithelial neoplasia, and cervical cancer [27], which suggested that HPV could be an important risk factor in disease progression. Here, we described the profile of HPV types in specimens from pregnant women with and without vaginitis which revealed the presence of 16 different HPV types, including the high-risk and low-risk mucosal types such as HPV16 and HPV6 which were detected in both pregnant women with and without vaginitis. Beside the high-risk and low-risk mucosal HPV types, many non-mucosal HPV types were also detected in the vaginal tract of pregnant women with vaginitis but not in pregnant women without vaginitis, suggesting these types of HPV might be associated with vaginitis.

Although anelloviruses are widely prevalent in human and animals and most appear to be commensal infections whose concentration increases with decreased immune function [28–30], there is no reports showing the presence of anellovirus in the vaginal tract of pregnant women with vaginitis. The present study revealed a large number of anellovirus sequence reads in the vaginal tract from pregnant women with vaginitis but not in the pregnant without vaginitis. The numerous anelloviral reads in the vaginal tract of vaginitis women may reflect the lower immunity status of these individuals or these types of anelloviral infection may have association with vaginitis of pregnant women. According to the criteria of International Committee on Taxonomy of Viruses (ICTV), demarcating species in genus *Betatorquevirus* employs a cut-off values of > 35% nucleotide sequence divergence based on full length ORF1, the novel anellovirus strains (with strain name ydyzj_2020 and GenBank no. MT783403) identified in this study are proposed novel species within the genus *Betatorquevirus*.

Human norovirus is a leading cause of acute viral gastroenteritis, resulting in significant morbidity worldwide [31]. Generally, norovirus infection has not been associated with extra intestinal manifestation and no report revealed the presence of norovirus in vaginal tract of pregnant women. In this study, noroviral sequence reads were also detected in vaginal tract of pregnant women with vaginitis, suggesting norovirus may have association with vaginitis, however, this should be verified by further research with large size of sampling.

Apart from these viruses fully characterized above, some herpesvirus sequences were also detected in all of the 6 libraries, however, some herpesvirus sequences were also present in the negative control library which included PBS as a sample for library constructing and the subsequent Miseq sequencing, suggesting some herpesvirus sequences in the vaginal libraries were from environmental contamination or experimental materials, these herpesvirus sequences were therefore not included in the results.

## Conclusion

Using viral metagenomics, the virome in vaginal tract secretion samples from pregnant women with and without vaginitis was investigated, which included 16 different types of HPV, 5 different species of anellovirus and norovirus. Genome sequences of 12 strains of HPV belonging to 10 different types and 5 different species of anellovirus were acquired and fully characterized. One of the novel anellovirus strains was proposed novel species within the genus *Betatorquevirus*. Our data laid a useful foundation for the future study of the correlation between virus community and vaginosis.

## Data Availability

The 6 sets of raw sequence data from the 6 viral metagenomic libraries were deposited in the Short Read Archive of GenBank database with BioProject accession number of PRJNA645620. The complete genome sequences of human papillomavirus and anellovirus which were fully characterized in this study were submitted to GenBank with the accession no.: MT783403- MT783419.

## Abbreviations

BLAST: Basic Local Alignment Search Tool
HPV: human papillomavirus
NCBI: National Center for Biotechnology Information
ORF: open reading frame
ICTV: International Committee on Taxonomy of Viruses
TTMV: TTV-like mini virus
NVNR: non-virus non-redundant
